# Quantitative PCR in soil-transmitted helminth epidemiology and control programs: Toward a universal standard

**DOI:** 10.1371/journal.pntd.0009134

**Published:** 2021-03-04

**Authors:** Piet Cools, Johnny Vlaminck, Jaco J. Verweij, Bruno Levecke

**Affiliations:** 1 Department of Virology, Parasitology and Immunology, Ghent University, Merelbeke, Belgium; 2 Laboratory for Medical Microbiology and Immunology, Elisabeth-Tweesteden Hospital, Tilburg, the Netherlands; University of Cambridge, UNITED KINGDOM

There is an increased interest to apply quantitative PCR (qPCR) in epidemiological studies and large-scale deworming programs targeting soil-transmitted helminths (STHs), due to important advantages over the current practice of microscopic stool examination (e.g., higher sensitivity and hookworm differentiation) [1]. Accordingly, qPCR should provide information on STH prevalence and infection intensity to evaluate progress toward WHO program goals (<2% moderate-to-heavy intensity infections) [2] and the ultimate goal to break transmission in targeted geographical areas [1,3]. However, qPCR is an umbrella term for a plethora of different procedures in which the results are most often expressed in units that do not allow interlaboratory comparison [4]. Reporting qPCR results in a universal unit that allows comparison is essential to standardize and compare protocols and subsequently implement qPCR in a programmatic decision algorithm. However, there is currently no consensus on such a universal unit, let alone on how to make international accepted standards available for the community.

In this viewpoint, we first delineate the ideal characteristics of such a universal unit (to be used in combination with universal standards, i.e., standardized material that contains a known amount of STH material) in an STH programmatic setting. We then provide a brief overview of the different units that are used to report qPCR results and discuss their benefits and limitations. Subsequently, we illustrate the use of genome equivalents per mL (GE/mL) as a potential universal unit. We end this viewpoint by proposing some necessary steps to pave the way toward a universal standard for nucleic acid amplification techniques in an STH programmatic setting. A discussion on other challenges for standardizing qPCR, such as the existence of different methods to preserve stool samples, DNA extraction, and qPCR protocols, each with their own efficiency, is not within the scope of the present viewpoint. For a more detailed discussion on these challenges, we refer to previously published work [4–8].

## The characteristics of an ideal universal qPCR unit for STH epidemiology and programs

A unit to report and analyze qPCRs results should meet 2 criteria:

1. the unit allows to compare quantitative results obtained in different assays (e.g., single versus multiple copy DNA targets) and/or by different laboratories; and
2. the unit can be aligned with program decision-making (e.g., classification of moderate-to-heavy intensity infections and assessing drug efficacy).

The first criterion can best be illustrated by the limit of detection (LOD or analytical sensitivity). The LOD is the minimum amount of target DNA that can be detected with a 95% certainty and is a key parameter to benchmark different diagnostic assays [9]. By definition, the LOD of a qPCR assay can only be determined and compared to other qPCRs only if results are expressed in quantitative units that allow interlaboratory comparison. Although the LOD is not an indicator that would be used in the STH programmatic context, it is a critical auxiliary parameter allowing programmatic evaluations and decisions to be made on a global scale. Once programs goals move from morbidity control toward elimination of disease, it will become important to determine and compare the LOD among protocols and ultimately identify a qPCR protocol that minimizes the probability of reporting false negatives. This is particularly important in populations where the infection intensity is in the same order of magnitude as the LOD. Using a qPCR with a high LOD would result in a lower prevalence and consequentially lead to a premature cessation of preventive chemotherapy programs. Also, the introduction of universal qPCR standards in an effort of global standardization would only be meaningful in case there is a unit that allows comparison of universal standards between laboratories. The second criterion implies that the results are expressed in a unit that allows an absolute quantification of infection of intensity. This, in turn, is important to assess both the prevalence of moderate-to-heavy intensity infections [2] and the therapeutic drug efficacy, which is based on the ratio of the infection intensity before and after treatment [10–11].

### Currently applied units to report qPCR results in the STH-field

#### Relative quantification units

**Fig 1:**
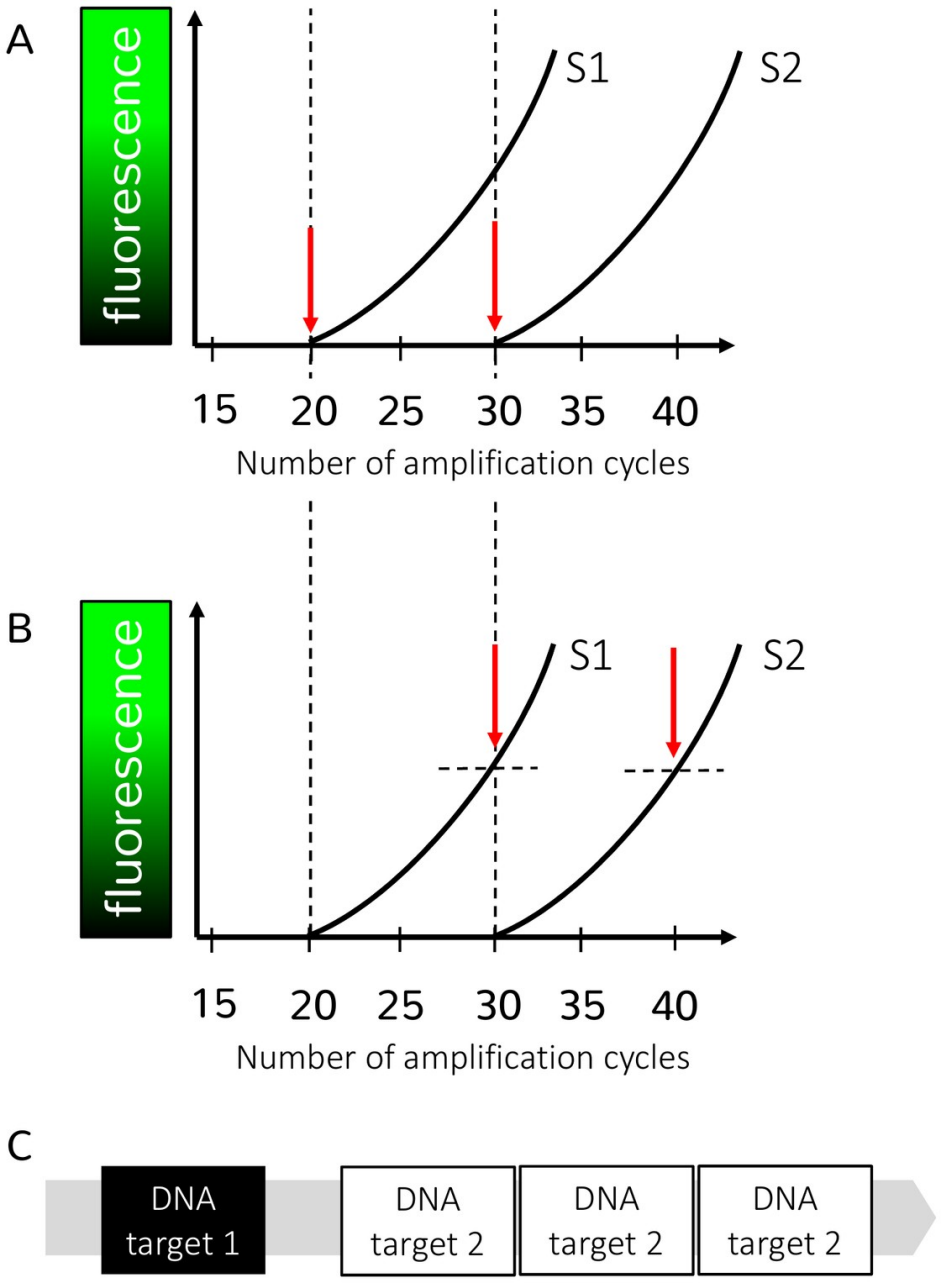
Challenges in relative and absolute quantification of DNA based on Cq units. Panels A and B describe the principles of and challenges in relative quantification of DNA, based on Cq units. The x-axis of these panels shows the number of amplification cycles, and the y-axis shows the level of fluorescence detected at each amplification cycle. The sooner a signal is observed, the higher the initial DNA concentration. To illustrate the effect of 2 qPCR platform-specific approaches to determine the Cq value, the same 2 amplification curves of 2 samples (S1 and S2) are depicted in panels A and B. In panel A, the Cq values are defined as the point where the fluorescence signal becomes detectable from the background (y = 0), and the amplification curves have Cq values of 20 and 30 for sample S1 and S2, respectively. This way of determining Cq values is, for example, used by software of the LightCycler480 qPCR platform (Roche). In contrast, in panel B, Cq values are defined as the point where the amplification curve crosses a threshold (dashed horizontal lines), and the same samples as in panel A have a Cq value of 30 (S1) and 40 (S2). This way of determining Cq values is used, for example, in the software of the StepOnePlus qPCR platform (Applied Biosystems). The principle of relative quantification is also shown in panels A and B. In both panels, the difference between the Cq values of the 2 samples is 10 (Cq 30 − Cq 20 in panel A and Cq 40 − Cq 30 in panel B). Therefore, in both panels A and B, S1 contains approximately 1,024 (2^difference in Cq^ = 2^10^ = 1,024) times more DNA than S2. Although in both panels this relative quantification of both samples is the same, the Cq values of the same samples differ substantially across the different platform-specific approaches. Furthermore, the absolute quantity of DNA is unknown, rather, it describes the quantity in samples relatively to each other (relative quantification). Panel C describes the challenges in absolute quantification of DNA based on Cq units. The gray bar represents a simplified genome of an STH species (e.g., *Trichuris trichiura*). DNA target 1 is a single-copy target, meaning that it is present only once in the whole genome. DNA target 2 is a multi-copy target, meaning that this target is present multiple times in the genome. As an example, laboratory A uses a single-copy gene as a DNA target to detect and quantify *T*. *trichiura* and reports a result of 100 genes/mL DNA extract. Laboratory B uses a multi-copy gene as a DNA target and reports a result of 300 genes/mL DNA extract. Because of the unknown quantitative relationship between these 2 genes in the *T*. *trichiura* genome, it is also unknown if both laboratories report the same *T*. *trichiura* DNA concentration. If both laboratories would express their qPCR results in GE/mL of DNA extract, both would theoretically find the same concentration of 100 GE/mL. Cq, cycle of quantification; GE/mL, genome equivalents per mL; qPCR, quantitative PCR; STH, soil-transmitted helminth.

Target DNA is doubled during each qPCR amplification cycle, and this amplification of target DNA results in the generation of a fluorescence signal. The rise of a fluorescent signal (above the background fluorescence) indicates amplification, and hence the presence of target DNA (Fig 1, panels A and B). The cycle of quantification (Cq) is the consensus name for the cycle number when fluorescence is detected above the background signal. Although the calculation differs, threshold cycle (Ct) or crossing point (Cp) values represent the same measurement [4] (see Fig 1). The Cq is inversely correlated with the initial concentration of DNA target in samples (less cycles are needed when more target DNA is present). As target DNA is doubled each cycle, the Cq scale is logarithmic in nature, and negative samples remain undefined, as there will never be a cycle where fluorescence will be above background.

The Cq unit is not appropriate as a universal unit as it does not meet the 2 criteria. First, in contrast to what is often thought, Cq values do not allow a quantitative interlaboratory comparison. Indeed, Cq values obtained from the same samples may largely vary across qPCR protocols, even if exactly the same target DNA is amplified. For example, different Cq values of the same samples can be obtained when the qPCR protocols differ in chemistry (qPCR mix; primer sequence and concentration; probe sequence, concentration, and fluorescent signal; and magnesium concentration), platform, and the way the Cq value is calculated. The latter is illustrated in more detail in panels A and B of Fig 1. Consequently, Cq values can only be used for relative quantification of DNA targets within the same assay in one particular laboratory. The results of a recent external quality assessment scheme, where a panel of DNA and stool samples were analyzed by 15 different laboratories across the world, clearly illustrated a very broad range in Cq values [4]. Second, the Cq unit does not provide information on the absolute quantity of target DNA and only represents the detection of amplification. Therefore, it would not allow for an absolute quantification of infection of intensity.

#### Absolute quantitative units

To determine the absolute DNA or egg concentration using qPCR assays, one needs to create a standard curve (= calibration curve) of Cq values obtained from standards with known target concentrations (e.g., target DNA incorporated in plasmids constructs [12] or DNA extracted from a known number of eggs [11,13]). Cq values from unknown samples are compared to the standard curve, in order to determine the DNA or egg concentration. For a DNA-based calibration, this results in units such as mass of DNA per volume (e.g., femtogram DNA per μl), DNA copy numbers per volume [12], whereas for an egg-based calibration, this results in either absolute egg counts or number of eggs per gram of stool [13]. Yet, each of these units have some important disadvantages. The DNA-based units do not allow comparison across qPCR protocols using different DNA targets [4,14]. This is mainly because it remains unknown how many copy numbers of each DNA target are present in the genome (the genomes of STH have not yet been fully annotated, and as a consequence of this, it remains unknown how a DNA concentration of target A compares to a DNA concentration of target B in qPCR (Fig 1, panel C). Although this can be largely avoided by an egg-based calibration, the egg-derived units too are not ideal. First, it generally conflicts with the practice to express an assay in a unit that reflects its analyte (DNA versus eggs). Moreover, although the reasoning of an egg-based calibration is to directly align with fecal egg counts, it has been shown that qPCR estimated egg counts not always correspond with the observed fecal egg counts [13], warranting an additional calibration step to fully align with program decision-making. Finally, there are some practical obstacles to make and distribute standards based on eggs, including but not limited to the availability of large number of eggs for each of STH species, the labor-intensive process to both purify and pick and place eggs, and the varying stages of egg development.

Standard curves can also be generated using genomic DNA extracted from worms [15]. This allows to express qPCR results in GE/mL, an absolute quantitative DNA unit that elegantly overcomes the issues described above. This unit (GE/mL) has already been applied extensively to report absolute quantities of many different bacterial (e.g., tuberculosis [16]), viral (e.g., Severe Acute Respiratory Syndrome Coronavirus 2 (SARS-CoV-2) [17]), and protozoan infections (e.g., malaria [18]), but only recently for STHs [4,10]. Moreover, genomic DNA is, once available, much easier to aliquot and hence ideal to ensure a worldwide distribution.

### qPCR results expressed in GE/mL in an STH programmatic setting

Recently, the methodology to express qPCR results in GE/mL and to determine the LOD for STHs has recently been described and applied [4,15]. The correlation of STH DNA and fecal egg counts is already known [6,8,19,20], and recently, categorization into classes of infection intensity (low versus moderate to high, a key indicator for decision-making) was shown to be possible when applying qPCR expressed in GE/mL, using qPCR specific thresholds for infection intensities [21]. Results were in good agreement (Fleiss–Cohen kappa statistic ranging from 0.49 to 0.70) with the Kato–Katz reference method for categorization [21]. Similarly, it was shown that the reduction in GE/mL following drug administration resulted in therapeutic efficacy estimates that were almost identical to those measured as the reduction in egg counts using the Kato–Katz thick smear [10].

### The way forward

Although the aforementioned studies highlight the potential of GE/mL as a universal unit for qPCR in STH epidemiology and control programs, some important challenges remain. There is a need to validate the use of GE/mL as a universal unit. In a proof-of-concept study, a set of DNA standards should be distributed globally to a selection of laboratories that subsequently assess the LOD of their in-house assays and verify if equal quantitative results are obtained based on standard curves expressed in GE/mL. A next step would be to ensure worm material (material from expulsion studies in humans or maintained in animal models, e.g., *Necator americanus*), centralize genomic DNA and, ultimately, to distribute them to a wide international network. One organization that already distributes such DNA standards for the standardization of nucleic acid tests is WHO [22]. WHO not only provides international standards for nucleic acid tests targeting viruses (e.g., hepatitis B virus, hepatitis C virus, and HIV) and bacteria (e.g., *Mycoplasma* spp.), but also for parasites such as *Plasmodium falciparum* and *Toxoplasma gondii*. Including standards for the different STH species would be one step forward toward further standardization of qPCR protocols in the STH field. Meanwhile, continued efforts to complete the annotation of STH genomes can open the pathway for the use of plasmids as standards, which could be a more cost-effective and feasible standard compared to worm material.

In conclusion, there is a clear need for more standardization in reporting qPCR results in order to use qPCR in STH epidemiology and control programs. One crucial aspect is the need for universal qPCR STH standards expressed in a universal unit that allows comparison of qPCR results across laboratories and qPCR protocols and the translation of these results into program decision algorithms. Further studies should focus on how to organize and validate the production and distribution of DNA standards for the STH community.
